# Sex Differences in Body Composition Changes after Preseason Training in Elite Handball Players

**DOI:** 10.3390/ijerph17113880

**Published:** 2020-05-30

**Authors:** Ireneusz Cichy, Andrzej Dudkowski, Marek Kociuba, Zofia Ignasiak, Anna Sebastjan, Katarzyna Kochan, Slawomir Koziel, Andrzej Rokita, Robert M. Malina

**Affiliations:** 1 Department of Team Sport Games, University School of Physical Education in Wrocław, Al. I. J. Paderewskiego 35, 51-612 Wroclaw, Poland; ireneusz.cichy@awf.wroc.pl (I.C.); andrzej.dudkowski@awf.wroc.pl (A.D.); andrzej.rokita@awf.wroc.pl (A.R.); 2 Department of Physical Education, Military University of Technology, ul. gen. Sylwestra Kaliskiego 2, 00–908 Warsawa, Poland; marekkociuba@wp.pl; 3 Department of Biostructure, University School of Physical Education in Wroclaw, Al. I. J. Paderewskiego 35, 51-612 Wrocław, Poland; zofia.ignasiak@awf.wroc.pl (Z.I.); kkochan2@wp.pl (K.K.); 4 Department of Anthropology, Hirszfeld Institute of Immunology and Experimental Therapy, Polish Academy of Sciences, ul. Rudolfa Weigla 12, 53-114 Wrocław, Poland; slawomir.koziel@hirszfeld.pl; 5 Department of Kinesiology and Health Education, University of Texas, Main Building (MAI), 110 Inner Campus Drive, Austin, TX 78705, USA; rmalina@1skyconnect.net

**Keywords:** handball, athletes, bioelectrical impedance, preseason training

## Abstract

The purpose of this study was to evaluate changes in the estimated body composition of elite female and male Polish handball players during a five-week preseason training camp. Height and weight were measured, while body composition was estimated with bioelectrical impedance in 18 male and 17 female handball players before and after the five-week training protocol. Components of body composition included total body water (TBW), fat-free mass (FFM), muscle mass (MM), and absolute and relative fat mass (FM). Weight and body mass index (BMI) did not change in males, but declined in females after five weeks of training. FM and %FM declined, while estimated TBW, FFM, and MM increased significantly after training in both males and females. In contrast, comparisons of log transformed ratios for changes in weight, the BMI and body composition in males and females, respectively, suggested that estimated TBW, FFM, and MM increased relatively more in females than in males, while FM and %FM decline relatively more in males than females. Overall, the five-week preseason training program modified the body composition of male and female handball players. FM and %FM decreased, while estimated TBW, FFM, and MM increased, in both males and females after the preseason training program. Comparisons of log transformed ratios for changes in body composition in males and females suggested sexual dimorphism in response to intensive preseason training.

## 1. Introduction

Body composition is often viewed as central to success in sport at many levels [1]. Although the two-compartment model of body composition—body weight = fat-free mass (FFM) + fat mass (FM)—was used in many early studies of body composition among athletes, often with a specific focus on estimates of relative FM (FM%), body composition can be approached at several levels [1], and advances in technology *per se* and methods have facilitated assessment so that FFM *per se* and lean tissue mass (LTM) and bone mineral content (BMC) or bone mineral density (BMD) components can be readily estimated, in addition to FM [2,3]. Among methodological developments, dual energy X-ray absorptiometry (DXA) and bioelectrical impedance analysis (BIA) are increasingly used in studies of body composition among athletes [4,5,6]. Nevertheless, no single method is the “gold standard”. 

The demands of specific sports vary considerably, and must accommodate variation associated with the extremes of size and mass in some sports and/or positions within a sport. As such, attention is commonly focused on endurance sports [7]; strength; speed and power sports [8]; and weight-sensitive sports, i.e., sports associated with low adiposity, fluctuation in weight, weight categories, dieting, and/or disordered eating [9]. Team sports are often included among strength, speed, and power sports, but must accommodate the demands of specific positions within a sport and the overall demands of the sport, e.g., size and mass variation in American football by position, height *per se* in basketball, etc. In addition, attention is often focused on the changes in body composition associated with training for sport in children and adolescents [10,11] and in young adults [12]. 

Estimates of the body composition of handball players have been largely based on several anthropometric protocols [13,14,15,16,17,18,19,20] that are associated with a considerable degree of variability, e.g., inter- and intra-technician measurement variability *per se* [21] and errors associated with specific prediction equations [12]. In contrast, estimates for handball players based on current technology, specifically BIA and DXA, are limited at present [22,23,24]. Nevertheless, many of the studies are largely descriptive and comparative, while few address training effects.

Although training methods have changed over time, often with a focus on specialization, expectations of athletes and coaches may differ relative to body mass and composition, especially in the context of absolute and relative fatness. Moreover, changes in estimated body composition prior to a season are of interest as both players and coaches prepare for the new season. In the context of the preceding, the purpose of this study is to evaluate changes in the estimated body composition of elite female and male Polish handball players during a five-week preseason training camp.

## 2. Materials and Methods

The study was conducted according to the Declaration of Helsinki, and the project was approved by the Research Bioethics Committee of the Faculty Senate of the University School of Physical Education in Wrocław, Poland (No. 4/2020; adopted in 05/03/2020; Chairperson prof. Marek Mędraś). The participants were elite Polish handball players (MKS Zagłębie Lubin Handball Team, Super Liga), 17 women (27.5 ± 4.5 years) with 16±4 years of experience in the sport, and 18 men (24.2 ± 3.3 years) with 12 ± 5 years of experience in the sport. All athletes provided written informed consent to have body composition estimated on the first and then on the final day of a five-week preseason training program, i.e., prior to the competitive season. 

### 2.1. Anthropometry and Body Composition

All athletes were measured under similar conditions in the morning (10 a.m. to 1 p.m.) and afternoon (2 and 5 p.m.) before and after the preseason training program (see below). Measurements consisted of body height (anthropometer, GPM, Zurich, Switzerland) and body weight (InBody 230 system, Tanita, Tokyo, Japan). The body mass index (BMI, kg/m^2^) was calculated. Based on BIA, InBody 230 system provides estimates of body composition, specifically total body water (TBW), fat-free mass (FFM), lean body mass (LBM), muscle mass (MM), and absolute and relative fat mass (kg, FM; %FM). 

### 2.2. Training Protocol

The training program for both males and females began six weeks before the start of the Polish Superliga season. The training cycle spanned 36 days and included similar daily routines for men and women, with 5 and 4 free days for women and men, respectively. The program included 56 training sessions and 12 control games for women, and 50 training sessions and 9 control games for men. Training sessions were conducted outdoors, in the gymnasium or indoor arena, and/or swimming pool, and were held twice per day. Sessions were specifically focused on the development of endurance, static and dynamic strength, speed, individual techniques, and tactics, and included internal controlled games and time for recovery. About 38% and 48% of training time was devoted to techniques and tactics in women and men, respectively. Estimated intensities of the training sessions are summarized in Table 1. The camp concluded seven days before the start of the league season.

### 2.3. Analysis 

Descriptive statistics (means, standard deviations, and medians) were calculated for body size and components of body composition before and after the preseason training program. Sex-specific Student t-tests for dependent groups were initially calculated to evaluate the significance of changes associated with training. Changes in each component of body composition were also calculated as ratios using the following formula: log^2^ (post-training/pre-training value). Transformation of the raw values at each observation has two advantages: (1) pre- and post-training changes in body composition are independent of pre-training levels in each individual, and (2) changes in different components are in turn comparable between sexes and among individuals since they are expressed as ratios and not as absolute or relative units. The transformed ratio was compared to a “0” value for changes in each component separately for each sex. 

In addition, sex-specific zero order, rank order (Spearman rho), and partial correlations, controlling for age and for both age and age squared, between preseason indicators of body mass and composition, and change scores after the training program were also calculated. Given the age range of the samples (females 19–38 years, males 19–36 years), age *per se* is an important covariate, and age-squared controls for the nonlinear distribution of age in the samples.

## 3. Results

Body mass and the BMI did not change among males, while body mass increased slightly but significantly and the BMI did not change among females after the five-week training program. In contrast, the specific components of body composition changed significantly after the training program (Table 2). FM and %FM declined significantly, while estimated TBW, FFM, and MM increased significantly after training in both males and females. Consistent with other studies, FM and %FM were higher in females than males, while TBW, FFM, and MM were higher in males than females. 

The transformed values for changes in body mass, the BMI, and specific components of body composition from pre- to post-training for males and females are presented in Table 3. Body weight and the BMI did not change, while all estimates of body composition changed significantly with intensive training among males. In contrast, all variables changed significantly with intensive training in females. In both sexes, MM and FFM increased, and FM and %FM decreased with training. 

Comparisons of the transformed ratios for change in weight, BMI, and body composition in males and females, respectively (Table 3, far right column), suggested that estimated TBW, FFM, and MM increased relatively and significantly more in females than in males, while FM and %FM declined relatively more, though not significantly so, in males than females. By inference, training-associated changes in weight and the BMI of males were associated with a decline in fatness and a moderate increase in FFM and MM, while corresponding training-associated changes in weight and the BMI among females were associated primarily with an increase in FFM and MM and a moderate decline in fatness.

Correlations between pre-training body weight and composition and subsequent changes after five weeks of intensive training are summarized in Table 4. Among males, changes in FM and %FM were significantly but negatively correlated with pre-training levels of fatness, i.e., players with higher levels of fatness at baseline experienced greater declines in FM and %FM after the training program. Among females, only the partial correlation for body weight is significant, while the other correlations for weight and those for FM are similar in magnitude but not significant. The direction of the correlations for females suggests that players with higher levels of body weight and absolute fatness tended to show larger declines in weight and FM after the training program.

## 4. Discussion

The results highlight changes in body composition associated with an intensive preseason training program among elite female and male handball players. On average, absolute and relative fat mass declined significantly and the lean components of body mass increased significantly. The direction of changes was generally consistent with expectations of coaches and athletes. In contrast, changes in body mass associated with the five-week training program were negligible and not significant in males, and relatively small but significant in females. The BMI was not affected by the preseason training protocol, which was consistent with the literature and highlighted the limitations of the BMI with among athletes. In males, mean BMIs before and after the training program (Table 2) were in the lower range of overweight (25.0 ≤ BMI < 30.0 kg/m^2^), which likely reflected the significant development of and gains in lean tissue mass associated with training. In females, on the other hand, mean BMIs were within the normal range and changed negligibly with training. Consistent with the literature [1,6,21,25], the findings highlight the limited utility of the BMI in female and male handball players.

Studies of changes in body composition associated with systematic training have a relatively long history [1,11,12], and results of the present study of female and male handball players were generally consistent with the literature, although corresponding studies of handball players using current technology are limited. A decline in FM, estimated with BIA, was noted among adolescent female handball players following eight weeks of high-intensity interval training, whereas players following a standard training protocol showed no significant change in FM [22]. Compared to a sample of 85 female players from six teams participating in the Italian national championship, the sample of Polish players (Table 2, based on BIA) was, on average, older, taller, and heavier, and had a lower FM of 13.7 kg, compared to 16.7 kg in the Italians sample, which was based on DXA [23].

As noted earlier, many studies of handball players have focused on characteristics of players by level of competition and by position, and on comparisons of handball players with athletes in other sports. The current sample of male players was, on average, younger, shorter, and slightly lighter than the Polish team participating in the 2013 Handball World Championship [26]. Elite male handball players in several studies were taller and heavier, had a higher lean tissue mass, and had a lower fat mass, than players at lower competitive levels [13,14,15]. The estimates of body composition in the comparative studies were based on anthropometry. Among females, elite players were, on average, taller and heavier, and had a considerably lower %FM and a higher lean tissue mass, especially of the upper limbs (based on DXA), compared to sub-elite players [24]. A similar trend was also apparent among junior (16 years) top elite, elite and non-elite handball players, although body composition was estimated anthropometrically [16].

The heights, weights, and anthropometric estimates of body composition of female and male handball players from several teams participating in the 12th Asian games (1994, Hiroshima, Japan) were compared [17,18]. Among 60 female players, differences among players from China, Japan, Kazakhstan, and South Korea were relatively small, but players from China were tallest with a larger estimated muscle mass, while those from Japan were shortest, lightest, and lowest in estimated FM [17]. In the comparison with male players, those from China, Japan, and South Korea (labeled East Asia) were, on average, slightly taller and heavier than players from Kuwait and Saudi Arabia (labeled West Asia), while predicted %FM was slightly less and predicted muscle mass was higher in the East than the West Asian players [18]. Among the East Asian male players, those from China were significantly taller, while those from South Korea were lighter.

Several studies of male handball players suggested variation in body size by position [17,18,19,20,21,22,23,24,25,26,27,28,29,30]. Wings were, on average, shortest with one exception, while backs tended to be tallest, though not in all samples; pivots, though not much shorter than backs, were intermediate. For body weight, pivots tended to be heaviest with backs slightly lighter; wings, in contrast, were lightest. Overall, there was considerable overlap between backs and pivots. Although data are less extensive, body size showed similar variation by position among female handball players [24]. It is likely that the size and the demands of specific playing positions in handball contribute to variation in body composition and regional variation in body composition (especially in the upper limbs) among players by position [28]. This is particularly noticeable in female and male pivot players, where greater muscle mass facilitates direct competition with the defender, allowing, perhaps, for a better position prior to throwing at the goal. 

Compared to elite male basketball players, elite male handball players were, on average, shorter and lighter, while differences in anthropometric estimates of relative muscle, bone, and fat between players in the two sports were relatively small [31]. Among male junior players (16–17 years) in four sports, volleyball and basketball players were, average, tallest heaviest, followed by handball and then soccer players [32]. In contrast, anthropometric estimates of muscle and bone content overlapped considerably among players in the four sports, while handball players had, on average, a higher estimated fatness than players in the three other sports. Among national level Greek female athletes, volleyball players were, average, tallest, followed by basketball and then handball players. In contrast, the handball players had a higher anthropometric estimate of FM than players in the other two sports [20]. 

Allowing for variation in body size *per se* and variation among methods for estimating body composition, it is likely that sport-specific demands, especially by position, and training protocols contribute to variation in body composition among athletes in the respective sports. Handball is a sport that involves frequent contact with opponents and many high-intensity actions. Knowledge of the physical characteristics of players, including body composition, may assist in the identification individual aptitudes of athletes, which in turn may affect behaviour and effectiveness during play, and perhaps facilitate a profile for sports training [18].

## 5. Summary

Weight and BMI did not change during the preseason training program in males, but declined in females. Estimated MM and FFM increased, while FM and %FM decreased after training in both males and females. The absolute increases in TBW and MM and the declines in FM and %FM with five-weeks of intensive training were slightly greater in males than in females. However, comparisons of log-transformed ratios for changes in weight, BMI, and body composition in males and females, respectively, suggested that estimated TBW, FFM, and MM increased relatively more in females than in males. By inference, there appeared to be a sexual dimorphism in response to intensive preseason training. 

The study is not without limitations. The duration of the preseason training program was limited to five weeks. This was a decision of the coaches. In addition, the number of players was relatively small, which limited the utility of comparisons of players by position.

## Figures and Tables

**Table 1:** Number of training sessions by intensity as noted by the coaches of the respective teams.

	Low	Moderate	High	Submaximal	Maximal
Women	1	13	27	6	9
Men	1	15	20	6	7

**Table 2:** Descriptive statistics (means [M], standard deviations [SD], and medians [Md]) for body mass and estimates of body composition among male and female handball players before and after training, and results of t-tests comparing pre- and post-training measures.

	Pre-Training			Post-Training			
	Mean	SD ^#^	Median	Mean	SD	Median	t
**Males (*n* = 18)**							
Height, cm	188.8	4.9	187				
Weight, kg	92	9.8	92.1	91.9	10.2	91.7	0.5
BMI, kg/m^2^	25.8	2.1	25.1	25.7	2.2	25.1	0.54
TBW, kg	60	5.4	59.9	61.1	6	61	3.49 *
FFM, kg	81.9	7.4	81.7	83.4	8.3	83.3	3.57 *
FM, kg	10.1	4.6	9.4	8.5	4	7.3	4.13 *
FM, %	10.8	4.1	10.5	9.1	3.6	8.4	4.34 *
MM, kg	47.2	4.4	47.1	48.1	4.9	48.3	3.63 *
**Females (*n* = 17)**							
Height, cm	176.3	7	176.2				
Weight, kg	70.6	8.9	69	71.4	8.6	70	3.70 *
BMI, kg/m^2^	22.6	1.7	22.2	22.9	1.6	22.4	3.73
TBW, kg	41.7	4	40.8	43.4	4	42.8	11.02 *
FFM, kg	56.9	5.4	55.6	59.3	5.5	58.5	11.18 *
FM, kg	13.7	4.1	12.6	12.1	3.9	11.9	8.25 *
FM, %	19.1	3.6	18.9	16.7	3.6	16.6	10.24 *
MM, kg	31.9	3.1	31.5	33.3	3.1	33	11.17 *

* *p* < 0.01; ^#^ standard deviation.

**Table 3:** Transformed values (M, SD) changes in body mass, body mass index (BMI), and estimates of body composition after the five week training program among male and female handball players and results of the t-tests. The transformed values are compared relative to "0". Results for the comparison of transformed values of males and females are also indicated along with results of t tests for independent samples).

	Males (*n* = 18)			Females (*n* = 17)			
log2(t_2_/t_1_) of	M	SD	t	M	SD	t	t Sex Comparison
Weight	−0.31	2.26	−0.58	1.62	1.74	3.84 **	−2.28 **
BMI	−0.31	2.26	−0.58	1.62	1.74	3.84 **	−2.82 **
TBW	2.43	2.90	3.55 **	5.77	2.23	10.67 ***	−3.80 **
FFM	2.43	2.85	3.62 **	5.89	2.27	10.71 ***	−3.95 **
FM	−25.69	23.11	−4.72 ***	−18.77	9.20	−8.42 ***	−1.15
%FM	−25.06	21.81	−4.87 ***	−20.44	8.93	−9.44 ***	−0.81
Muscle	2.65	3.08	3.64 **	6.52	2.58	10.44 ***	−4.02 **

** *p* < 0.01; *** *p* < 0.001.

**Table 4:** Correlations between pre-training values and respective changes (deltas) in weight and components of body composition after five weeks of training.

	Males (*n* = 18)			Females (*n* = 17)		
	Zero Order	Partial ^†^	Spearman	Zero Order	Partial ^†^	Spearman
Weight, kg	0.22	0.31	0.1	−0.37	−0.44 **	−0.30
Muscle, kg	0.37	0.27	0.34	−0.10	−0.28	−0.21
FFM, kg	0.41 **	0.31	0.28	−0.00	−0.17	−0.07
FM, kg	−0.52 *	−0.57 *	−0.50 *	−0.36	−0.35	−0.27
FM, %	−0.47 *	−0.54 *	−0.44	−0.09	−0.12	−0.11

^†^ Partial correlations controlling for age and age2, * *p* ≤ 0.05, ** *p* ≤ 0.10.
